# Usability Analysis of a Health Sciences Digital Library by Medical Residents: Cross-sectional Survey

**DOI:** 10.2196/23293

**Published:** 2021-06-24

**Authors:** Amr Jamal, Shabana Tharkar, Hanan Alenazi, Bedoor Saud Julaidan, Dania Ali Al Hindawi, Norah Suleman AlAkeel, Ola Mohammed AlNuhayer, Raneem Hamoud AlDubaikhi

**Affiliations:** 1 Evidence-Based Health Care & Knowledge Translation Research Chair, Department of Family and Community Medicine College of Medicine King Saud University Riyadh Saudi Arabia; 2 Prince Sattam Chair for Epidemiology and Public Health Research, Department of Family and Community Medicine College of Medicine King Saud University Riyadh Saudi Arabia; 3 National Health Information Center Saudi Health Council Riyadh Saudi Arabia; 4 College of Medicine King Saud University Riyadh Saudi Arabia

**Keywords:** digital library usability, medical education, system usability scale, medical residents, Saudi Arabia

## Abstract

**Background:**

The usability of a digital library depends on a myriad of factors ranging from the end users’ ability to website complexity. Although digital libraries provide instant access to online content, offering an efficient reference platform, their usability is highly variable.

**Objective:**

The aim of this study was to measure users’ perspectives and usability of the digital library of the Saudi Commission for Health Specialties (SCFHS).

**Methods:**

A web-based questionnaire survey was conducted using a validated System Usability Scale (SUS) containing 5 positive and 5 negative items on the usability of the digital library. The SUS standard cut-off score of 68 was considered for interpretation.

**Results:**

The overall mean SUS score of digital library usability was 52.9 (SD 15.2) with a grade “D” categorization, indicating low usability. The perceived measures of attributes of the 10 SUS items of findability, complexity, consistency, and confidence obtained below average scores. Only item 1 relating to perceived willingness to use the digital library frequently obtained a score above the targeted benchmark score (mean score 3.6). Higher SUS scores were associated with training (*P*=.02). Men felt the digital library to be more complex (*P*=.04) and board-certified physicians perceived a greater need for training on digital library use (*P*=.05). Only the UpToDate database was widely used (72/90, 80%).

**Conclusions:**

These findings demonstrate the low usability of the extensive facilities offered by the SCFHS digital library. It is pivotal to improve awareness of the availability of the digital library and popularize the databases. There is also a need for improved user training to enhance the accessibility and usability of the multiple databases.

## Introduction

The transition of conventional print materials to electronic formats has revolutionized the use of libraries. Digital libraries are a result of the marvelous technological advancements of the 21st century. The availability of extensive online literature has curtailed the classic style of print-based articles and photocopying, resulting in diminished usage over the last decade [1]. Digital libraries provide comprehensive databases containing electronic books, journals, online gray literature, web publishing, and evidence-based guidelines and literature, with additional benefits of animation, audio-visual aids, and mobile apps with scientific content. Institutional libraries are an invaluable asset in making users competent in terms of scientific knowledge, research skills, and keeping pace with the most updated information [2]. As early as the second half of the 1990s, when internet technology was still in a nascent stage, Morse and Clintworth [3] compared the usage of a traditional library with the then-novel electronic library (e-Library) and found an astoundingly high predominance of electronic usage among health science students of an academic library in California. Recent studies have demonstrated that the worldwide web has made traditional libraries obsolete, forcing students to rely entirely on digital libraries even in low- and middle-income countries [4,5]. Given that the economics of establishing and running a digital library costs millions of dollars, its quality of services and effectiveness should be decisive and significant.

The Saudi Commission for Health Specialties (SCFHS), which was established in 1983, is the regulatory body in Saudi Arabia that is responsible for the country’s health care workforce in aspects related to licensing, training, and continuous professional development. The SCFHS has been commissioned as the Kingdom’s largest and most competent digital library service, facilitating the work of health care practitioners and trainees from all specialties, thereby empowering continuing medical education [6]. The SCFHS extensively partners with international institutions, and subscribes to leading medical and health care journals and online databases. Residents are given free access to the digital library by registering with a valid residency ID. The provision of such extensive facilities and universal access to online literature necessitates the end users’ usability description. The term “usability” has varied definitions since this multidimensional construct can be evaluated from different perspectives. Some authors have linked usability to ease of use, considering the effectiveness of human-computer interaction [7], while others prefer the usability definition highlighted by Brinck et al [8] “as the degree to which people (users) can perform a set of required tasks.” The perceived usability of interactive systems is evaluated to identify usability issues, improve the usability of the design, and to encourage recommendations [9].

Although a library is a platform that offers access to a variety of databases and applications, the library portal itself consists of many features and services that warrant assessment of their usability measures. The user experience includes the start of accessing the digital library portal until acquiring the needed information. This journey involves many interactions with the portal, such as the universal search, finding the right database/journal/book, authenticating access to third-party portals, technical and librarian support, and integration with other systems. All of these user-portal interactions are related to the library itself and warrant usability assessment. This study was performed with the objective of assessing the usability of the digital health sciences library provided by the SCFHS among trainees, including their information-seeking behavior and perceptions in the usability of the digital library. The findings may point toward the generic deficiencies in any digital library, which, if rectified, may enhance the user interface and increase the usability of the digital library for professional development in research to ameliorate evidence-based practice**.**

## Methods

### Study Design and Sampling

A quantitative cross-sectional study design was applied to assess the usability of the digital library of the SCFHS. The study participants included the trainee residents from all specialties with valid registration at the SCFHS. A list containing the registered names of 3455 professionals was obtained from the SCFHS, which constituted the sample frame. Assuming that 50% of the population conveys positive perceptions of digital library usability, with a 95% CI and a precision of 5%, the estimated sample size using the single proportion formula was calculated to be 384. This study was performed over a period of 2 months, in March and April of 2018. The study tool was constructed using the SurveyMonkey platform. The tool was sent online to all registered email IDs listed in the sample frame. Two reminder emails were sent after 2 weeks to increase the response rate.

### Description of the Questionnaire

This study adopted the validated questionnaire of the System Usability Scale (SUS) developed by John Brooke in 1996 [10]. The SUS system is a robust, reliable, and valid tool, which has been extensively used by researchers. It has also shown a high degree efficiency in testing library systems, repositories, and websites [11]. The first section comprises 10 questions measuring the system usability of various hardware, software, websites, and applications, with scaled responses on a 5-point Likert scale ranging from strongly disagree to strongly agree. The SUS is considered to be an easy-to-use tool with simple and straightforward statements arranged as odd numbers (1, 3, 5, 7, 9) that are positively expressed and statements with even numbers (2, 4, 6, 8, 10) that are negatively expressed.

The 10 items on the SUS are as follows: (1) I think that I would like to use this system frequently, (2) I found the system unnecessarily complex, (3) I thought the system was easy to use, (4) I think that I would need the support of a technical person to be able to use this system, (5) I found the various functions in this system to be well-integrated, (6) I thought there was too much inconsistency in this system, (7) I would imagine that most people would learn to use this system very quickly, (8) I found the system very cumbersome to use, (9) I felt very confident using the system, and (10) I needed to learn a lot of things before I could get going with this system.

The sequence of the statements was created so as to reduce concurrence and extreme bias. The second section comprised questions on demographic characteristics, accessibility, training and experiences in using databases, and suggestions to improve the digital library. A pilot study was performed with 12 participants to ensure reliability and validity of the questions in section 2, and these responses were not included in the main analysis.

### Scoring SUS Responses

The SUS scores were calculated with reference to the guidance outlined by Jeff Sauro [12]. The scores for each question were converted to a new number, summed, and then multiplied by 2.5 to convert the original scores ranging from 0 to 40 to a range of 0 to 100. The SUS scores were interpreted with a cutoff of 68. A score above 68 indicates average performance, whereas a score below 68 is considered to be below average. Furthermore, comparison and easy reference of individual items of findability, complexity, consistency, and confidence can be achieved using targeted benchmark scores that make the responses more meaningful [12].

### Data Analysis

The data were analyzed using the IBM software Statistical Package for Social Sciences version 22.0. Descriptive statistics (frequencies, percentages, mode, and mean and SD) were used to describe the categorical and quantitative variables. Testing of associations between categorical and continuous variables was performed by the Student *t* test and analysis of variance, respectively. A *P* value less than .05 was judged to indicate statistical significance.

### Ethical Considerations

The study was reviewed and approved by the Institutional Review Board Committee of College of Medicine, with reference number CMED 305-F8-2018-18. All participants were informed about the purpose of the study and electronic informed consent was obtained on the first page of the survey tool. The study ensured that the participants’ data will be confidential, private, and used for research purposes only.

## Results

Ninety completed questionnaires were received at the end of the study period. The majority of the respondents were men with a mean age of 29.3 years (SD 4.2). The distribution of demographic characteristics is provided in Table 1.

The study participants’ overall average SUS score was 52.9. The average mean score in comparison with benchmark scores for the 10 SUS items are presented in Table 2. Item 1, which states “I think that I would like to use the digital library frequently,” was the only SUS item that obtained a score above the targeted average benchmark.

The participants’ experience with the SCFHS digital library is shown in Table 3. The most successful method of spreading awareness of the SCFHS digital library was email. However, communication through colleagues was also popular. UpToDate was the most predominantly used database, whereas Cochrane and DynaMed showed low utilization. The most frequently stated purpose for use of the SCFHS digital library was preparation for presentations, closely followed by patient care-seeking information. The use of the digital library for research and teaching support ranked in the third and fourth priority, respectively. The other popular alternatives to the SCFHS digital library were the participants’ respective institutions’ libraries, followed by the Saudi Digital Library.

The associations between demographic and other variables with the total score are presented in Table 4. The results showed that training on how to access databases was significantly associated with a higher total score (*P*=.02). Although not significant, female gender, non-Saudi nationality, and senior residency levels obtained higher SUS scores. However, the analysis of individual SUS items with different study variables showed two items with a significant association. The score for item 2, which states “I found the digital library to be unnecessarily complex,” was significantly higher in men than in women (*P*=.04) and among board-certified physicians (*P*=.01). The score for item 4, which states “I think that I would need the support of a technical person to be able to use this digital library,” was significantly higher among board-certified physicians (*P*=.05).

**Table 1:** Demographic characteristics and System Usability Scale (SUS) scores of Saudi Commission for Health Specialties digital library users (N=90).

Variable		Value
**Gender, n (%)**		
	Male	53 (59)
	Female	37 (41)
**Nationality, n (%)**		
	Saudi	83 (92)
	Non-Saudi	7 (8)
**Age (years), n (%)**		
	20-25	8 (9)
	26-30	57 (63)
	31-35	20 (22)
	>35	5 (6)
**Residency level, n (%)**		
	R1	17 (19)
	R2	27 (30)
	R3	14 (16)
	R4	11 (12)
	Fellowship	6 (7)
	Board-certified	3 (3)
**Medical specialty, n (%)**		
	Family medicine	16 (18)
	Internal medicine	17 (19)
	Pediatrics: general	11 (12)
	Surgery	9 (10)
	Psychiatry	3 (3)
	Radiology	4 (5)
	Emergency medicine	3 (3)
	Community medicine/public health	3 (3)
	Critical care medicine (intensive care unit)	3 (3)
	Other	9 (10)
**SUS score**		
	Mean (SD)	52.9 (15.2)
	Range	25-100

**Table 2:** Comparison of the mean scores with benchmark targets for average scores of the 10 System Usability Scale items.

Item	Benchmark target mean score	Obtained mean score
1: I think that I would like to use digital library frequently	≥3.39	3.6
2: I found the digital library to be unnecessarily complex	≤2.44	2.8
3: I thought the digital library was easy to use	≥3.67	3.3
4: I think that I would need the support of a technical person to be able to use the digital library	≤1.85	3.1
5: I found the various functions in the digital library to be well-integrated	≥3.55	2.9
6: I thought there was too much inconsistency in the digital library	≤2.20	2.9
7: I would imagine that most people would learn to use the digital library very quickly	≥3.71	3.3
8: I found the digital library to be very cumbersome to use	≤2.25	3.0
9: I felt very confident using the digital library	≥3.72	3.1
10: I needed to learn a lot of things before I could get going with the digital library	≤2.09	3.2

**Table 3:** Participants’ experience with the Saudi Commission for Health Specialties (SCFHS) digital library (N=90).

Question		Participants, n (%)
**How did you learn about the SCFHS digital library?**		
	Colleagues	21 (23)
	Email	59 (66)
	Trainers	7 (8)
	Twitter	5 (6)
	WhatsApp	7 (8)
	SCFHS website	4 (4)
	Others	5 (6)
**What are the databases that you used within the digital library?**		
	UpToDate	72 (80)
	Cochrane	14 (16)
	Clinical evidence	17 (19)
	Best evidence	14 (16)
	DynaMed Plus	19 (21)
	Others	13 (14)
**Purpose (s) for use of the SCFHS digital library?**		
	Research	62 (69)
	Support teaching activities	55 (61)
	Patient care	68 (76)
	Preparation for presentation	70 (78)
	Preparation for examination	46 (51)
	Continuing medical examination	35 (39)
	Others	2 (2)
**What other electronic library/libraries do you have access to?**		
	None	32 (36)
	Saudi Digital Library	37 (41)
	My institute/hospital/center library	43 (48)
	Others	10 (11)

**Table 4:** Association of questionnaire responses with System Usability Scale (SUS) scores (N=90).

Question		Response, n (%)	Total SUS score, mean (SD)	*P* value
**Do you pay to use any other databases or electronic libraries?**				.40
	Yes	18 (20)	50.2 (15.8)	
	No	72 (80)	53.5 (15.0)	
**Did you have previous experience with electronic libraries before the SCFHS^a^ digital library?**				.94
	Yes	61 (68)	52.8 (16.6)	
	No	29 (32)	53.1 (12.3)	
**Have you ever received training on how to access and use health information databases/libraries?**				.02
	Yes	20 (22)	59.5 (14.5)	
	No	70 (88)	51.1 (14.5)	
**Gender**				.35
	Male	53 (59)	51.8 (13.8)	
	Female	37 (41)	54.9 (17.3)	
**Nationality**				.51
	Saudi	83 (92)	52.8 (14.5)	
	Non-Saudi	7 (8)	56.7 (25.5)	
**Residency level**				.08
	R1	17 (22)	53.2 (8.7)	
	R2	27 (35)	51.1 (17.4)	
	R3	14 (18)	59.4 (14.2)	
	R4	11 (14)	51.5 (17.4)	
	Fellowship	6 (8)	66.6 (17.7)	
	Board-certified	3 (4)	39.1 (10.1)	
**Residency category**				.28
	Junior (R1 and R2)	44 (49)	51.9 (14.6)	
	Senior (R3 and R4)	25 (28)	56.0 (15.8)	
**Age group (years)**				.99
	20-25	8 (9)	51.5 (9.8)	
	26-30	57 (63)	53.2 (15.7)	
	31-35	20 (22)	53.3 (16.9)	
	>36	5 (6)	53.0 (17.6)	

^a^SCFHS: Saudi Commission for Health Specialties.

## Discussion

### Principal Findings

This study focusing on the usability of the SCFHS digital library by registered trainee health practitioners is the first of its kind in Saudi Arabia. Some of the main findings indicate that digital library use in terms of perceived complexity, consistency, and confidence is below average among the trainees. Since the overall SUS score obtained in this study was 52.9, a grade D categorization may be given, denoting low usability [13]. Benchmarking of the scores of individual SUS items provides useful interpretation [14]. In this study, item 1 that reflects the digital library’s frequent use was the sole item that obtained a score above the benchmark score. This finding suggests the participants’ increased perceived usability toward using the digital library, whereas the rest of the SUS items obtained low scores, suggesting difficulty in all attributes of measures of complexity, consistency, and confidence.

Nevertheless, on a positive note, the SUS score showed a significant increase among those who attended a voluntary introductory training session on an ad hoc basis that was provided either by the individual databases or by their affiliated institutions through webinars and YouTube sessions. However, it must be emphasized that no formal training sessions were organized facilitating use of the digital library. These key findings may draw attention. Research studies have linked high computer literacy and social media to increased digital library usability among users. Umukoro et al [15] examined the factors associated with increased digital library use among university students by performing a mixed methods study in Nigeria, and established three major predictors among the users. They linked high system and service quality, computer skills, and level of satisfaction with electronic services to increased e-Library use, whereas the determinants of not using the digital library were primarily lack of awareness of services, inadequate computer skills, and absence of user-training facilities. These results are highly consistent with our findings, which could indicate the chief reasons for low usability. Similarly, Piccoli et al [16] built a conceptual framework of contributing factors for effective electronic learning (e-Learning) that depends on the interface between human and design factors. Human factors constitute the instructors’ and students’ roles such as motivation, learning, and training, whereas design factors include technology, course contents, and an interactive environment that have a direct effect on the level of use. A qualitative analysis demonstrated incomprehensible website design and content as some of the major themes for low usability of a digital library, which suggested that the search protocol should be established in accordance with the end users’ expectations to improve website usefulness [17]. Since some users may demonstrate computer literacy, the reasons for low usability remain ambiguous. In-depth qualitative research is required to comprehend the factors associated with low SUS scores. The poor system usability may reflect a combination of factors or attributes that regulate actual usability.

The other main finding of training-assisted improvement in digital library usability suggests that the respondents may enhance usability with essential training skills and operative assistance. These findings are in accordance with similar studies from Nigeria and Zimbabwe, where lack of training and awareness were identified as principal barriers for poor electronic resource utilization [4,5,18]. These findings are further supported by a study that demonstrated improved e-Learning behavior after implementation of a successful end-user internet literacy training program for university students [19]. Our study thereby suggests the definite need for training measures to be reinstated by the SCFHS for the effective use of digital library facilities. Future studies obtaining pre and post evaluation measures of efficacy of training programs are highly recommended.

Another issue of concern is the lack of awareness of digital library facilities. The finding that only 3.7% of the participants knew about the digital library facility through the SCFHS website is alarming. The content display on the website plays a constructive role in spreading awareness about the digital library. There is an immediate need to exemplify content guidance to improve awareness and accessibility of the digital library in the SCFHS website. Hinchliffe and Mummery [20] elucidated the significance of the involvement of intended audience and end users in designing websites through usability testing to improve and optimize user experiences. Likewise, we recommend performing similar research to obtain users’ suggestions for content directions of the SCFHS website highlighting the digital library facilities, which can result in increased usability.

Furthermore, the library usage patterns demonstrate that preparation for presentations and obtaining information on routine patient care are the two most common reasons for use. Although these results are reasonably expected from the trainees, the suboptimal usage for other relevant purposes warrants further clarification. The use of a digital library during examinations and for research and additional support materials was found to be low. However, the use of alternate sources such as institutional libraries and the Saudi Digital Library may account for the probable low use. These findings are strong indicators of the suboptimal awareness of available online databases. A similar study performed at a university library in the United States showed that 94.5% of residents accessed the resources for patient care, 92% of nursing professionals accessed the library for class preparations, and 76% accessed the library for research purposes [1]. There is great potential for research to further discover the reasons for low usage among the participants. Library orientation for residents and fellows, web helpdesk service, training on online database searching skills, and web-based notification systems are some of the suggested methods to improve usability [21].

This study found an inclined preponderance toward the use of UpToDate as the most popular online database. This is also reflected in the lower utilization of other multiple databases. Provision of training facilities ought to incorporate content description and specifications of other online databases such as Cochrane, Best evidence, and DynaMed to maximize benefits. These three evidence-based medicine databases containing full free texts and voluminous literature on clinical trials with a level of evidence and recommendations based on established guidelines serve as priceless assets for both research and clinical practice. The reported low use of these resources raises concerns. Moreover, this may lead to concealing a significant amount of variance in perceived usability scores. These findings point toward the need for further exploratory research to identify the determinants and reasons for low usage, which can inform exhaustive measures to be taken to improve awareness and usability.

### Limitations

This study has certain limitations. The relatively small sample size restricts the generalizability of the findings. Furthermore, the sample included only trainees, and other health care providers who could have served as potential resource participants were not included, which again may lead to limited generalizability. However, the strength of this study is that it is the first such investigation in the region of Saudi Arabia, and the multiple findings generated can be considered as a vital basis for future research. Another relevant point is the ambiguity in the usability of each of the multiple online databases. This study assessed the overall usability of the digital library; however, details of the usability of individual databases were not explored, which can be a subject of future research.

### Conclusion

The concept of digital libraries has revolutionized the usability of libraries by providing instant and simplified access. The digital library of the SCFHS is a major source of referencing for health professionals in Saudi Arabia. This study is the first to evaluate the usability of the digital library of SCFHS.

Lower usability scores were obtained, demonstrating the below-average utilization of resources. The participants demonstrated low confidence in accessing the digital library, and needed technical support, guidance, and training programs for efficient access and use of the contents. The online database UpToDate was the most popular database accessed for clinical support decisions, whereas the remaining databases showed limited usability. However, training and orientation were associated with higher usability scores, suggesting a promising solution to improve usage. A well-structured training program facilitating simplified and efficient navigation of the digital library contents is the need of the hour. In addition, the SCFHS website should consider increasing awareness through innovative methods to increase the potential use of multiple databases. There is a great potential for modification in website design to increase awareness and improve digital library usability.

### Recommendations to Improve e-Library Usability

This research may highlight certain general recommendations to improve the usability of the SCFHS digital library based on the identified findings in two major contexts: personnel and technical. Primarily, at the outset, we highly recommend facilitating an end-user training program as a mandatory apprenticeship featuring the navigatory steps in addition to the introduction of the available online databases and their specifications for wider use.

Next, the focus of recommendations may include the technicality and design of the website. Accessibility can be markedly improved by direction and guidance in a user-friendly mode. In addition, the design should facilitate categorization and description of the digital library contents. The homepage of the website must contain a downloadable user-friendly guide as an overview of digital library orientation. Furthermore, the trainees must be assisted with provision of helpdesk facilities, online chats, and regular notifications on digital library updates.

## Abbreviations

e-Learning: electronic learning
e-Library: electronic library
SCFHS: Saudi Commission for Health Specialties
SUS: System Usability Scale

## References

[1] De Groote SL, Dorsch JL (2003). Measuring use patterns of online journals and databases. J Med Libr Assoc.

[2] Hassan B, Mansor Y (2009). Chapter 6. Role of academic libraries in promoting information literacy among students of higher learning institutions. Strengthening higher education for a successful workforce.

[3] Morse D, Clintworth W (2000). Comparing Patterns of Print and Electronic Journal Use in an Academic Health Science Library. Issues Sci Technol Librariansh.

[4] Martinez-Silveira MS, Oddone N (2008). Information-seeking behavior of medical residents in clinical practice in Bahia, Brazil. J Med Libr Assoc.

[5] Mawere T, Sai KO (2018). An investigation on e-resource utilisation among university students in a developing country: A case of Great Zimbabwe University. SA J Inf Manag.

[6] Saudi Commission for Health Specialties.

[7] Jeng J (2005). What is usability in the context of the digital library and how can it be measured. Inf Technol Librar.

[8] Brinck T, Gergle D, Wood S (2001). Usability for the web, designing websites that work.

[9] Conyer M (1995). User and usability testing - how it should be undertaken?. Austral J Educ Technol.

[10] Brooke J, Jordan PW, Thomas B, Weerdmeester BA, McClelland IL (1996). SUS: A QuickDirty Usability Scale. Usability Evaluation in Industry.

[11] Sauro J (2011). Measuring usability with the sytem usability scale.

[12] Sauro J (2018). Interpreting single items from the SUS.

[13] Brandy K (2017). An overview of the System Usability Scale in library website and system usability testing. J of Lib User Experience.

[14] Lewis J, Sauro J (2018). Item benchmarks for the System Usability Scale. J Usab Stud.

[15] Umukoro IO, Tiamiyu MA (2016). Determinants of e-library services’ use among university students: A study of John Harris Library, University of Benin, Nigeria. J Librariansh Inf Sci.

[16] Piccoli G, Ahmad R, Ives B (2001). Web-based virtual learning environments: a research framework and a preliminary assessment of effectiveness in basic IT skills training. MIS Quart.

[17] Kous K, Pušnik M, Heričko M, Polančič G (2018). Usability evaluation of a library website with different end user groups. J Librariansh Inf Sci.

[18] Adeniran P (2013). Usage of electronic resources by undergraduates at the Redeemer’s University, Nigeria. Int J Libr Inf Sci.

[19] Adeleke A, Olorunsola R (2010). Training in the use of e-resources in academic libraries: one university's approach. Library Hi Tech News.

[20] Hinchliffe A, Mummery WK (2008). Applying usability testing techniques to improve a health promotion website. Health Promot J Austr.

[21] Davies K, Harrison J (2007). The information-seeking behaviour of doctors: a review of the evidence. Health Info Libr J.

